# Genome‐Wide Analysis of the lncRNAs and miRNAs Involved in Flower Development in Radish

**DOI:** 10.1002/pld3.70155

**Published:** 2026-02-18

**Authors:** LingJun Wu, Xiaobo Luo, Yadong Li, Yueyue Jin, Wanping Zhang

**Affiliations:** ^1^ College of Agriculture Guizhou University Guiyang Guizhou China; ^2^ Institute of Vegetable Industry Technology Research Guizhou University Guiyang China; ^3^ Guizhou Institute of Biotechnology Guizhou Province Academy of Agricultural Sciences Guiyang China

**Keywords:** DEGs, flower, lncRNA, miRNA, radish, regulatory pairs

## Abstract

Radish ( *Raphanus sativus* L.) is an important root vegetable in the cruciferous family. The yield and quality of radish is seriously affected by the premature bolting and flowering. Although the microRNAs (miRNAs) in regulating flower development have been established in radish, the identification and characterization of long noncoding RNAs (lncRNAs) have yet to be explored. In this study, miRNAs and lncRNAs in vegetative and flower stage were conducted by RNA‐seq and small RNA sequencing, respectively. A total of 5315 differentially expressed genes (DGEs), 263 DElncRNAs, and 38 DEmiRNAs were detected in two stages. GO analysis found that many flower DGEs associated with reproductive process, response to hormone, and pollination were enriched. In total, 202 DElncRNAs and 257 DElncRNAs were found to have potential cis‐ and trans‐regulatory effects on 572 DEmRNAs and 3902 DGEs, respectively. A total of 93 and 82 DEGs were predicted as putative targets of 31 DEmiRNAs and 29 DEmiRNAs, respectively. Five mRNA‐lncRNA‐miRNA regulatory pairs involved in flowering time regulation were proposed, including miRNA156a‐5p, miRNA399b, miRNA novel‐23, miRNA164c‐5p, and miRNA165a‐5p. The qRT‐PCR results showed that four mRNAs, three lncRNAs, and three miRNAs were consistent with the results of RNA‐seq and small RNA sequencing. Transient overexpression of miR156a‐5p significantly inhibited the expression levels of *RsSPL10*, *RsSPL15*, lncRNAs RsLinc1162, and RsLinc214. The results showed that miR156 co‐expressed with *RsSPL10* and *RsSPL15* significantly inhibited the luciferase activity of *RsSPL10* and *RsSPL15* genes, indicating miR156 can directly target *RsSPL10* and *RsSPL15* and inhibit their expression. These findings provide a theoretical foundation for further elucidating the molecular regulation mechanism of mRNAs, lncRNAs, and miRNAs in bolting and flowering in radish.

## 1 Introduction

Long noncoding RNAs (lncRNAs) are usually longer than 200 nucleotides with no protein‐coding potential (Wu et al. 2017). LncRNAs are mainly transcribed by RNA polymerase II (Pol II), which are often capped, spliced, and polyadenylated (Xu et al. 2018). LncRNAs can be classified into long intergenic noncoding RNAs (lincRNAs), intronic lncRNA, and long noncoding natural antisense transcripts (lncNATs) based on their general genomic locations (Zheng, Chen, et al. 2019). Compared with protein‐coding genes, most lncRNAs are poorly conserved between species and exhibited low expression levels and tissue‐specific expression (Wang et al. 2016). Increasing number of studies established that lncRNAs are involved in diverse biological processes by modulating the expression of genes in a *cis* and *trans* manner (Ye et al. 2022). The lncRNAs were involved in chromatin remodeling, transcriptional regulation, and posttranscriptional regulation (Quinn and Chang 2016). Accumulating evidence suggests that plant lncRNAs play vital roles in flowering time regulation (Heo and Sung 2011), fruit development (Li et al. 2018), anthocyanin accumulation (Yang, Li, et al. 2019), biotic, and abiotic stress responses (Kindgren et al. 2018; Cui et al. 2019). In Arabidopsis, lncNAT *COOLAIR* and intronic lncRNA *COLDAIR*, transcribed from the *FLOWERINGLOCUS C* (*FLC*) locus, are required for epigenetic silencing of *FLC* by recruiting the polycomb repressive complex during vernalization process (Liu et al. 2010; Quinn and Chang 2016). A novel lncRNA *TaVRN1* promoted *TaVRN1* expression to accelerate flowering in winter wheat (Xu et al. 2021). LncRNA *MLNC3.2* and *MLNC4*.6 acting as eTMs for miR156a showed increased accumulation of anthocyanin in fruit peels in apple (Yang, Ma, et al. 2019). *MdLNC499* in apple peel was positively regulated the expression of *MdERF109* and promote anthocyanin biosynthesis (Ma, Chhapekar, et al. 2021). The lncRNA TRABA suppressed β‐glucosidase–encoding BGLU24 to promote salt tolerance in cotton (Cui et al. 2023).

Plant miRNAs are 22‐nucleotide noncoding RNAs that inhibit the corresponding target genes. The miRNAs play key roles in the regulation of flowering‐related processes, such as the juvenile‐adult transition, the induction of floral competence, and flower development (Hong and Jackson 2015). The miR172 as a translational repressor of *APETALA2* affected flower development in Arabidopsis (Chen 2004). Previous studies indicated that the miR156 and target genes *SPLs* in an *FT*/*FD*‐independent manner in regulating *Arabidopsis thaliana* flowering (Wang et al. 2009). Loss of Sly‐miR160 showed abnormal floral organ abscission and lateral organ lamina outgrowth in tomato (Damodharan et al. 2016). The FvemiR390a‐overexpressing plants exhibited delay flowering in woodland strawberry (Dong et al. 2022). The overexpression of miR399b and a loss‐of‐function allele of *PHO2* displayed an early flowering phenotype in *A. thaliana* (Kim et al. 2011). Overexpression of miR159 slightly accelerated vegetative development by affecting miR156 and *SPLs* in Arabidopsis (Guo et al. 2017). The miR166/165 cooperate with their target genes that are dynamically controlled in regulating the shoot apical meristem activity and floral development (Jung and Park 2007). Previous studies revealed that the miR164‐CUC2 module plays crucial roles in specifying leaf and floral organ morphology in strawberry (Zheng, Wei, et al. 2019). *TCP4* as a key target of miR319a is important for petal growth and development in Arabidopsis (Nag et al. 2009). These results demonstrated that various miRNAs involved in different flowering‐related phases of plant development, including the transition from juvenility to maturity, floral induction and development.

Radish is one of the important economic vegetables in the Asia. Bolting and flowering are important stages for plants to transition from the vegetative stage to the reproductive stage (Li et al. 2023). Radish takes fleshy roots as product organs, early bolting, and flowering in winter and spring or spring and summer will greatly affect the quality and commerciality of fleshy roots, resulting in the decline of economic benefits. In recent years, a series of advances have been made in the QTL mapping and gene function in radish bolting and flowering trait (Yi et al. 2014; Xu et al. 2023). Three radish *FLC* homologous genes *RsFLC1*, *RsFLC2*, and *RsFLC3* were identified, overexpression of these three genes in *A. thaliana* exhibited late‐flowering phenotypes, indicating that the three radish *RsFLCs* function as flowering suppressor (Yi et al. 2014). Transcriptome analysis of radish showed that 142 differentially expressed genes (DEGs) was involved in the physiological process of bolting and flowering (Nie et al. 2016). Based on transcriptome sequencing, 218 homologous flowering genes with *A. thaliana* were identified, of which 49 genes were differentially expressed, including *RsFLC*, *RsFT*, and *RsSOC1* (Jung, Lee, et al. 2016). Through sequence similarity and collinearity analysis with Arabidopsis and Cabbage, 254 flowering genes of radish were identified (Wang et al. 2017). QTL analysis found that *RsFLC2* was as the candidate gene for bolting trait and a 1627 insertion in the first intron of *RsFLC2* gene was closely linked with bolting trait (Wang et al. 2018). Two bolting QTLs and two flowering QTLs were identified in the constructed F_2_ population on chromosome R06 (Ma, Yang, et al. 2021). It was found that a 647‐bp insertion of *RsVRN1* gene promoter was directly bound by *RsCDF3*, and overexpression of *RsCDF3* showed delayed flowering in *A. thaliana* (Xu et al. 2023). However, the molecular mechanism of radish bolting and flowering remains unclear.

In this study, a late‐flowering radish “XHT” in vegetative growth and flower stage were used to identify and characterize differentially expressed mRNAs, lncRNAs, and miRNAs by RNA sequencing (RNA‐seq) and small RNA sequencing. The potential mRNA‐lncRNA‐miRNA regulatory network that might participate in the radish flower development was constructed. This study is helpful in understanding of roles of mRNA, miRNAs, and lncRNAs in the radish flower development and elucidating the potential regulatory mechanisms in bolting and flowering trait in radish.

## 2 Materials and Methods

### 2.1 Plant Materials and Growth Conditions

A radish high‐generation inbred lines “XHT” (late‐flowering time, 185 days) in vegetative growth and flower stage was used. The seedlings were planted in an artificial climate chamber (day temperature 25°C/16 h; night temperature 16°C/8 h; humidity 75%). Three true leaves were transplanted into plastic pots. The plants were conducted with 30 days of vernalization and transferred to a normal growth room and grown for flower. The samples were frozen with liquid nitrogen and then immediately stored in a refrigerator at −80°C degrees.

### 2.2 LncRNA Sequencing and Mapping

Total RNA was isolated from all samples using the TRIzol RNA Extraction Kit (Invitrogen, USA). After assessing the RNA purity, quantity and integrity, ribosomal RNA was removed by Epicentre Ribo‐zero rRNA Removal Kit (Epicentre, USA). Next, strand‐specific sequencing libraries were generated using the rRNA‐depleted RNA by NEBNext Ultra Directional RNA Library Prep Kit for Illumina (NEB, USA). The quality of the sequencing libraries was qualified by the Agilent Bioanalyzer 2100 system (Agilent Technologies, USA). Finally, the libraries were sequenced on an Illumina HiSeq 2000 platform and paired‐end 125‐bp reads were generated. Three independent biological replicates were prepared for each developmental stage for lncRNA sequencing.

The clean reads were obtained by removing adapter sequences, contaminating sequences, and low‐quality reads. The clean reads were subsequently aligned to the radish reference genome (https://ftp.ncbi.nlm.nih.gov/genomes/all/GCF/000/801/105/GCF_000801105.1_Rs1.0) using HISAT2 v2.0.4 (Liao et al. 2014). The mapped reads from each sample were assembled independently by StringTie (v1.3.3) (Pertea et al. 2015). All transcripts from each sample were merged to generate final transcripts using Cuffmerge (Trapnell et al. 2012).

### 2.3 Identification of lncRNAs and Differential Expression Analysis

To identify high‐confidence lncRNAs, the transcripts were subjected to a series of rigorous filtration steps. Transcripts with sequence length less than 200 bp and FPKM ≤ 0.5 were removed. The coding‐noncoding index (CNCI) (Sun et al. 2013) and Coding Potential Calculator (CPC) (Kong et al. 2007) was used to assess the coding potential of the transcripts. The transcripts containing known protein family domains were excluded by PfamScan (v1.3) (Punta et al. 2012). The transcripts were subjected to PhyloCSF (phylogenetic codon substitution frequency) software for examining evolutionary signatures characteristic to alignments of conserved coding regions (Lin et al. 2011). The remaining transcripts without coding potential were defined as candidate lncRNAs. The identified lncRNAs were classified into three categories according to their genomic localization.

FPKM (reads per kilobase per million reads) was employed to represent the normalized expression value. The FPKM of genes and lncRNAs were measured using StringTie software (Pertea et al. 2015). The edgeR software package was used to identify differentially expressed transcripts with *q* values < 0.05 and fold change ≥ 1 (Robinson et al. 2010).

### 2.4 Target Gene Prediction and Co‐Expression Networks Analysis

To determine the lncRNAs regulate protein‐coding genes in cis or in trans, the protein‐coding genes within 100‐kb upstream or downstream of the lncRNAs were analyzed. The expression correlation between lncRNAs and coding genes were calculated using Pearson correlation coefficient (PCC) values. The lncRNA‐mRNA pairs with a Pearson correlation > 0.95 and a *p* < 0.001 were defined as co‐expressed. Gene Ontology (GO) analysis of differentially expressed lncRNA target genes was carried out by the GOseq R package. GO terms with corrected *p* value < 0.05 were considered significantly enriched by differential expressed genes. Kyoto Encyclopedia of Genes and Genomes (KEGG) pathway enrichment analysis of lncRNA target genes were implemented by the KOBAS software (Mao et al. 2005).

### 2.5 Small RNA Sequencing, miRNA Identification, and Targets Prediction

The total RNA of six samples was extracted with TRIzol reagent according to the manufacturer's instructions. Small RNA sequencing in two libraries with three biological replicates were constructed using NEBNext Multiplex Small RNA Library Prep Set for Illumina and then sequenced on the Illumina HiSeq 2500 platform (Illumina) to generate 50‐bp single‐end reads (Novogene, Beijing, China). The adaptors and low‐quality reads were filtered out to obtain the clean reads. The clean reads with 18–30 nucleotides in length were mapped to the radish reference genome (https://ftp.ncbi.nlm.nih.gov/genomes/all/GCF/000/801/105/GCF_000801105.1_Rs1.0) by Bowtie (Langmead et al. 2009) without mismatch to analyze their expression and distribution. The known miRNA was identified using miRBase and modified miRDeep2 (Friedländer et al. 2012). Reads originating from known structural RNAs were removed by RepeatMasker and Rfam. The remaining small RNAs were used to predict and analyze novel miRNA by miREvo and miRDeep2 (Friedländer et al. 2012; Wen et al. 2012). The miRNA expression levels were estimated by TPM (transcript per million). The differentially expressed miRNAs were identified using the DESeq R package, and the threshold for significantly differential expression was a fold change of > 1.5 and a *p* value of < 0.05. The target gene of miRNA was predicted by psRNATarget (Dai et al. 2018). GO and KEGG enrichment analysis was conducted based on the target genes of differentially expressed miRNAs.

### 2.6 Transient Transformation of Radish Cotyledons

The 3320‐RUBY‐KAN vector with RUBY tag was constructed, and the vector was constructed by homologous recombination. The target gene construction vector was transformed into *Agrobacterium tumefaciens* (GV3101) competent cells, and the monoclonal was inoculated in the liquid LB medium containing the corresponding antibiotics. The shaking culture was carried out at 28°C and 200 rpm to OD600 = 0.8–1.0. The bacteria were collected by centrifugation at 4000 rpm for 10 min, resuspended with infection buffer (10 mmol/L‐1 MgCl_2_, 10 mmol/L‐1 MES, pH 5.6150 μmol/L‐1 AS), and induced at room temperature for 4 h. The radish cultivar “DY13” was cultured at 24°C (16 h light)/20°C (8 h dark) until the cotyledon fully expanded. The Agrobacterium suspension was slowly injected from the back of the cotyledon using a needle‐free syringe to spread the solution evenly to the entire cotyledon. Three biological replicates were set for each treatment. After injection, the plants were cultured in dark for 1 day, and then cultured under the original growth conditions. After 7–14 days, the cotyledon phenotype was observed and sampled for detection of mRNA stability.

### 2.7 Dual‐Luciferase Reporter Assay

A dual‐luciferase reporter assay was performed using the pGreenII 0800‐LUC and 62SK vectors to examine the regulatory effect of miR156 on *RsSPL10* and *RsSPL15*. The target fragments of *RsSPL10* or *RsSPL15* containing the predicted miR156 binding sites were cloned into the pGreenII 0800‐LUC reporter vector, in which firefly luciferase (LUC) was used as the reporter and Renilla luciferase (REN) served as an internal control. The miR156 effector construct was generated by cloning the miR156a sequence into the 62SK vector under the control of the CaMV 35S promoter, and the emptyp 62SK vector was used as a negative control. All constructs were transformed into *A. tumefaciens* strain GV3101 and infiltrated into fully expanded leaves of Nicotiana benthamiana. After infiltration, plants were grown under normal conditions for 48 h, and luciferase activities were measured using a dual‐luciferase reporter assay system according to the manufacturer's instructions. The relative LUC activity was calculated as the ratio of firefly luciferase activity to Renilla luciferase activity (LUC/REN). Each experiment was performed with at least three biological replicates.

### 2.8 Quantitative Reverse Transcription–PCR

The expression levels of DEGs, DE‐lncRNAs, and DE‐miRNAs were performed vegetative growth, six vernalization times (5, 10, 15, 20, 25, 30 days) and flower stage using qRT‐PCR. The experiment was performed with SYBR Premix Ex TaqTM Kit (Takara, Japan) using the CFX96 Real Time PCR system (Bio‐Rad, Hercules, CA, USA). The radish *Actin* was used as the internal control. All qRT‐PCR reactions were carried out with three biological replicates. The relative expression levels were calculated using the 2^−ΔΔCt^ method (Livak and Schmittgen 2001). The primers are listed in Table S1.

## 3 Results

### 3.1 Identification and Characterization of lncRNAs in Radish

**FIGURE 1:**
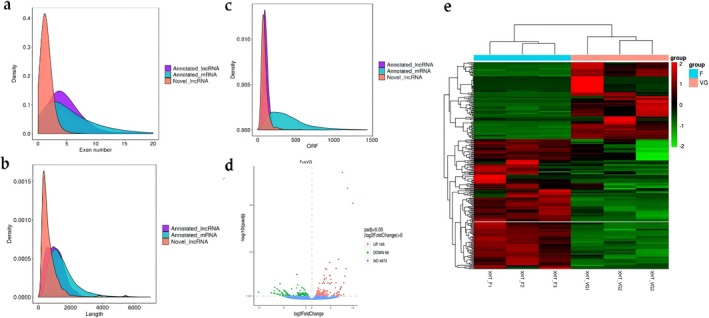
Identification and characterization of lncRNAs in radish. (a) Distribution of exon numbers in annotated lncRNAs, annotated mRNAs, and novel lncRNAs. (b) ORF (open reading frame) density distributions of lncRNAs, annotated mRNAs, and novel lncRNAs. (c) Length density distributions of lncRNAs, annotated mRNAs, and novel lncRNAs. (d) Volcano plot of all DElncRNAs in two stages. (e) Heat map of all DElncRNAs in two stages.

To systematically identify lncRNA expression profiles in radish, whole‐transcriptome strand‐specific RNA sequencing was conducted on two libraries with three biological replicates. A total of 255,975,102 and 266,418,206 clean reads were generated from XHT_VG and XHT_F, and over 90% reads were successfully aligned to the radish genome, respectively (Table S2). In total, 50,027 transcripts were obtained. After filtering the transcript, a total of 8828 lncRNAs were identified, of which 7299 were known lncRNAs (annotated on the radish genome) and 1529 were novel lncRNAs (new predicated except radish genome). Novel lncRNAs exhibit with fewer exons compared to annotated mRNAs and lncRNAs (Figure 1a). The sequence length of the lncRNA was substantially shorter than the mRNAs (Figure 1b). The ORF of the lncRNA was shorter than the mRNAs (Figure 1c). Expression analysis found that 263 lncRNAs were differentially expressed in XHT_VG compared with XHT_F, of which 165 were upregulated and 98 were downregulated (Figure 1d; Table S3). Hierarchical clustering analysis found that the differentially expressed lncRNAs exhibited tissue specificity between vegetative growth and flower stage in radish (Figure 1e).

### 3.2 Identification and Functional Enrichment Analysis of DEGs

**FIGURE 2:**
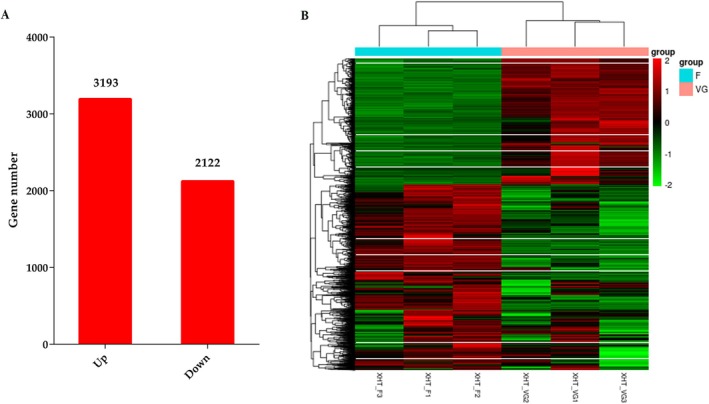
DGEs analysis in vegetative growth and flower stage of radish. (a) The number of upregulated and downregulated DGEs in two stages. (b) Heat map of all DGEs in two stages.

A total of 5315 DGEs were identified, of which 3193 DGEs were upregulated and 2122 were downregulated (Figure 2a, Table S4). The overall expression pattern of 5315 DEGs were obtained by clustering heatmap (Figure 2b). GO and KEGG enrichment analyses were performed to explore the potential functions of the DEmRNA. The DGEs were found to be significantly enriched in 52 GO terms (*p* < 0.05), including 30 terms under molecular functions, 14 terms under cellular components, and eight terms under biological processes (Table S5). The top 2 enriched GO terms in biological processes were related to photosynthesis and cellular carbohydrate metabolic process. The majority of the DGEs were associated with membrane protein complex and thylakoid under cellular components. In molecular functions, the majority of the DGEs were involved in the transferase activity and calcium ion binding. KEGG analysis showed that the DGEs were significantly enriched in 14 KEGG pathways, including carbon metabolism, biosynthesis of cofactors, photosynthesis, carbon fixation in photosynthetic organisms, and carotenoid biosynthesis (Table S6).

**FIGURE 3:**
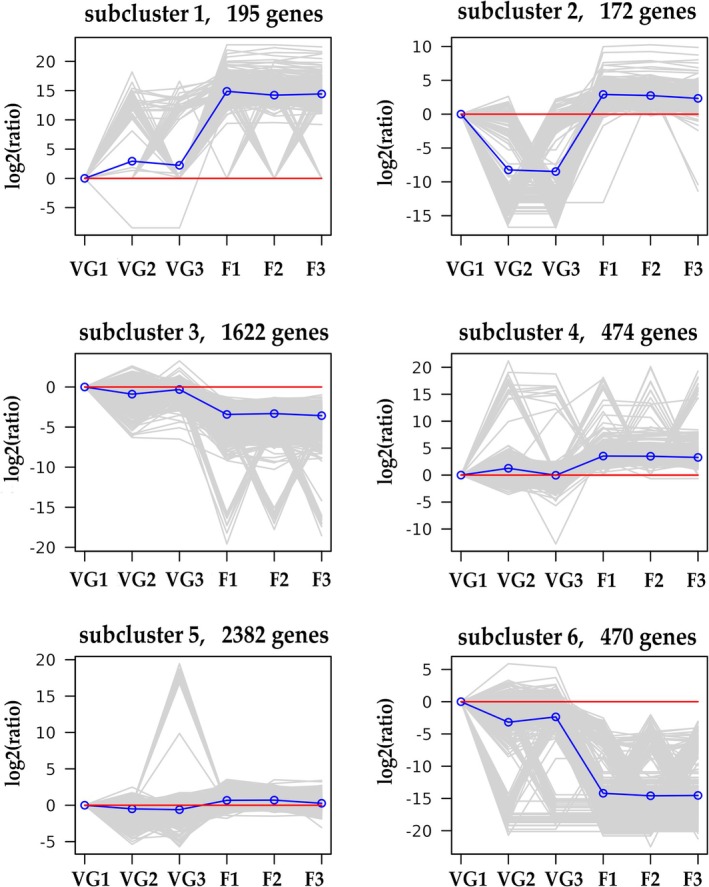
The expression patterns of DGEs in six clusters by K‐means algorithm.

To understand the expression patterns of all the DEGs, the expression data at two stages were subjected to k‐means clustering analysis. The results showed that all the DEGs were clustered into six clusters with distinct expression patterns (Figure 3). The DEGs in cluster 1 (195 DEGs), cluster 2 (172 DEGs), and cluster 4 (474 DEGs) were upregulated in flower stage. The DEGs in cluster 3 (1622 DEGs) and cluster 6 (470 DEGs) observed a similar expression patterns, and predominantly downregulated in the flower stage. The DEGs in cluster 5 (2382 DEGs) were no obvious differences in two stage. Many genes involved in flowering time regulation were observed in six clusters. GO analysis of the DEGs in cluster 1 related to developmental process, response to auxin, and response to hormone. This cluster included, *A GAMOUS‐like 18* (*AGL18*)‐like genes *FLOWERING LOCUS T (FT*), *EARLY FLOWERING* 3 (*ELF3*). The GO terms cell growth, catabolic process, and response to hormone were enriched in cluster 2. This cluster included, *SPL15, SUPPRESSOR OF OVEREXPRESSION OF CO 1* (*SOC1*), sugar transporter *SWEET11*‐like, and *SWEET12*‐like are responsible for regulating flowering time. The DEGs in cluster 3 were involved in the reproductive process and sucrose metabolic process. This cluster contained *FLOWERING LOCUS C* (*FLC*)‐like genes, *SQUAMOSA promoter‐binding protein‐like 4* (*SPL4*), PHYTOCHROME‐INTERACTING FACTOR 4 (PIF4), *AGL18*, *ELF4*, and *FLC5* that are important for flowering time regulation. In cluster 4, photosystem and photosynthetic membrane were the most significantly enriched GO terms. The photoperiod pathway genes *CONSTANS‐like 1* (*COL1*) in this cluster are responsible for regulating flowering time. The DEGs in cluster 6 was enriched in pollination, recognition of pollen, and response to hormone. These genes included *FLC*‐like genes, *AGL18*‐like genes, *EMBRYONIC FLOWER1* (*EMF2*), *calcium‐dependent protein kinase 6* (*CPK6*), flowering promoting factor 1 (*FPF1*), and *ERNALIZATION INSENSITIVE 3* (*VIN3*).

### 3.3 Overview of sRNA Sequencing Data and miRNA Identification

**FIGURE 4:**
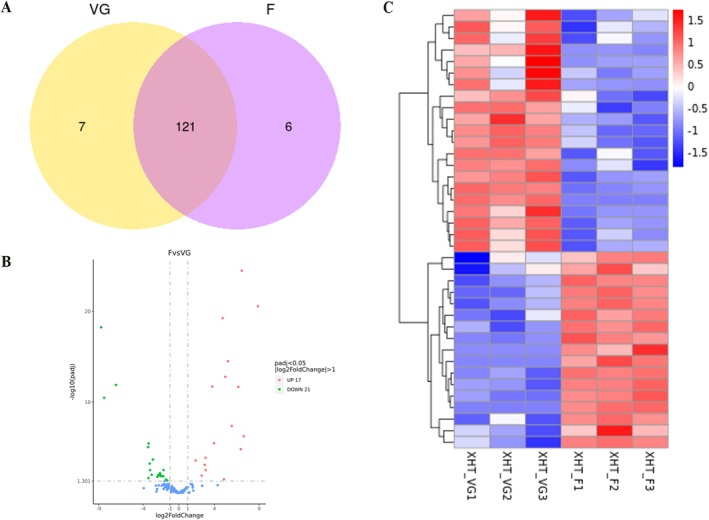
miRNAs analysis in vegetative growth and flower stage of radish. (a) Venn diagram of miRNAs identified in vegetative (VG) and flower stage (F). (b) Volcano plot of all DGEs in two stages. (c) Heat map of all DEmiRNAs in two stages.

To explore the miRNAs associated with flower in radish, two libraries with three biological replicates were constructed and sequenced from the leaves of XHT_VG and XHT_F. A total of 36,350,640 and 38,944,977 raw reads were generated in XHT_VG and XHT_F samples, respectively (Table S7). After removing adaptor sequences, low‐quality sequences, 35,462,073 and 38,092,343 clean reads were obtained in XHT_VG and XHT_F samples, respectively. In total, 134 miRNAs were detected, including 74 known miRNAs and 60 novel miRNAs. The length of the known miRNAs and novel miRNAs was ranged from 20 to 23 and 20 to 24 bp, respectively. A total of 121 common miRNAs were identified between XHT_VG and XHT_F samples, of which seven miRNAs were XHT_G specific and six miRNAs were XHT_F specific (Figure 4a). The differential analysis found that 38 miRNAs (21 known miRNAs and 17 novel miRNAs) were differential expressed in XHT_VG and XHT_F samples, of which 17 miRNAs were upregulated and 21 miRNAs were downregulated (Figure 4b, Table S8). The expression levels of DEmiRNAs exhibited obvious differences between XHT_VG and XHT_F samples (Figure 4c).

### 3.4 Prediction of Cis‐ and Trans‐Regulated Target Protein‐Coding Genes of lncRNAs

Cis‐regulated target genes of lncRNAs were predicted according to the position relationship between lncRNA and mRNA within a genomic window of 100 Kb. In total, 84 DElncRNAs were found to have potential cis‐regulatory effects on 74 DEmRNA in 263 gene pairs (Table S9). Among these, 17 DElncRNAs have one target DEmRNA and 57 DElncRNAs have more than one target DEmRNA. A total of 96 DEmRNAs were predicted as potential trans‐regulatory effects on 107 DElncRNAs in 25,520 gene pairs (Table S10). The correlation analysis found that a total of 81.83% positive correlation, while 18.17% negative correlation was found between lncRNAs and their potential target genes.

### 3.5 Target Genes Identification and Analysis

The DGEs and lncRNAs that possibly act as target of miRNA were identified by psRNATarget program. A total of 85 DEGs were predicted as putative targets of 31 DEmiRNAs in 89 miRNA‐target modules (Table S11). Correlation analysis indicated that 26 DEmiRNAs were negatively correlated with 45 DEGs in 47 miRNA‐target modules. GO annotation of the targets of DEmiRNAs showed that the majority of GO terms were associated with the nucleic acid binding, DNA binding and ion binding under MF, the biosynthetic process, regulation of cellular process, regulation of biological process, biological regulation under BP, and the membrane, organelle and cellular component under CC. KEGG pathway enrichment analysis showed most the targets of DEmiRNAs were involved in the biosynthesis of secondary metabolites pathway, purine metabolism and metabolic pathways. A total of 87 DElncRNAs were predicted as putative targets of 32 DEmiRNAs in 134 miRNA‐target modules (Table S12). Correlation analysis indicated that 26 differently DEmiRNAs were negatively correlated with 51 DElncRNAs in 67 miRNA‐target modules. The results showed that three miRNAs could potentially be targeted by one lncRNA and one miRNA could target 15 lncRNAs.

### 3.6 The mRNA‐LncRNA‐miRNA Regulatory Network of Flowering Regulation

**FIGURE 5:**
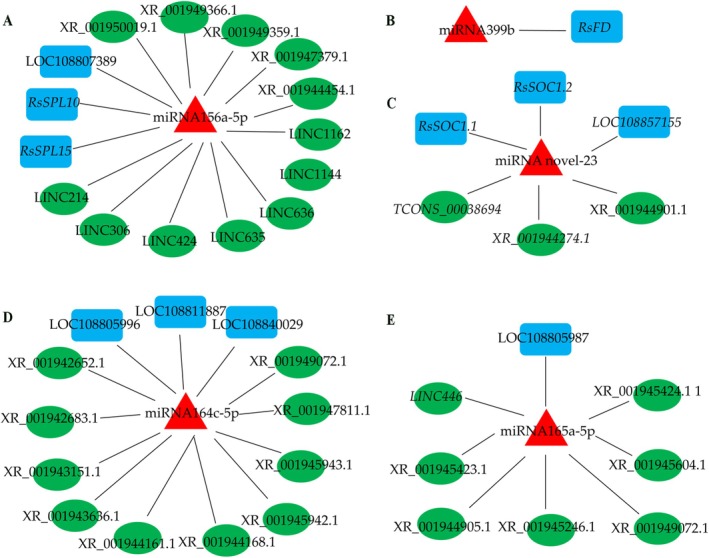
The lncRNA‐miRNA‐mRNA regulatory network. (a) miR156‐5p, three DEGs and 12 DElncRNAs. (b) miR399b and RsFD. (c) miRNA novel‐23, three DGEs and three DElncRNAs. (d) miR164c‐5p, three DGEs and 10 DElncRNAs. (e) miR165a‐5p, one DGEs and seven DElncRNAs. The blue square represents DEmRNA, green circle represents DElncRNA, and red triangle represents DEmiRNAs.

The miRNAs negatively regulated with their corresponding target genes were used to further analysis. The DEmiRNAs‐targeted DEGs associated with flowering pathway and DEmiRNAs‐targeted lncRNA co‐expression pairs were used to constructed the mRNA‐lncRNA‐miRNA regulatory pairs. The miR156a‐5p targeted with seven DEGs and 12 DElncRNAs, of which three DGEs were showed a negative correlation with miR156‐5p, including *SPL10* and *SPL15*. The three DEGs and 12 DElncRNAs were used to construct interaction network (Figure 5a). The *RsFD* gene and its negative regulator miR399b were performed to construct interaction network (Figure 5b). A mRNA‐lncRNA‐miRNA regulatory network contained miRNA novel‐23, targeted gene *RsSOC1*, and three DElncRNAs were constructed (Figure 5c). The miR164c‐5p–targeted three DGEs and 10 DElncRNAs were performed to construct interaction network (Figure 5d). The miR165a‐5p–targeted one DGE and seven DElncRNAs were used to construct interaction network (Figure 5e).

### 3.7 Validation of Gene Expression of mRNAs, lncRNAs, and miRNAs

**FIGURE 6:**
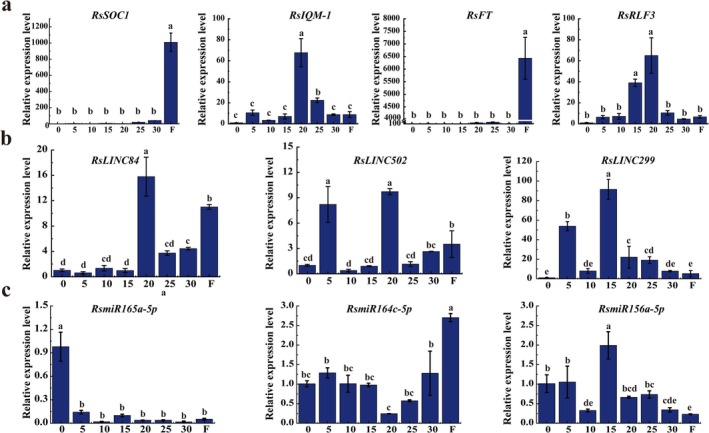
RT‐qPCR validation of DGEs, DElncRNAs and DEmiRNAs. (a) Relative expression levels of four DGEs. (b) Relative expression levels of four DElncRNAs. (c) Relative expression levels of three DEmiRNAs.

To verify the accuracy of the sequencing results, the relative expression levels of four genes, three lncRNAs, and three miRNAs were verified by qRT‐PCR. The results showed that the expression of *RsSOC1*, *RsIQM‐1*, *RsFT*, and *RsRLF3* were significantly upregulated in the flower stage, of which *RsIQM‐1* and *RsRLF3* were also significantly upregulated in six vernalization times (Figure 6a). Three lncRNAs, *RsLINC84*, *RsLINC502*, and *RsLINC299* were significantly upregulated in six vernalization times and flower stage (Figure 6b). *RsmiR399b* expression began to increase after 20 d of vernalization, reached a peak at 25 d, and remained at a relatively high level during the flowering stage. In contrast, *RsmiR169b‐3p* and *RsmiR156a‐5p* maintained overall high expression levels throughout the vernalization process but were downregulated after flowering (Figure 6c). These results were similar with the expression levels obtained by our RNA‐Seq and small RNA‐seq results.

### 3.8 Validation of the Role in miR156 and Its Targets in Radish Leaves

**FIGURE 7:**
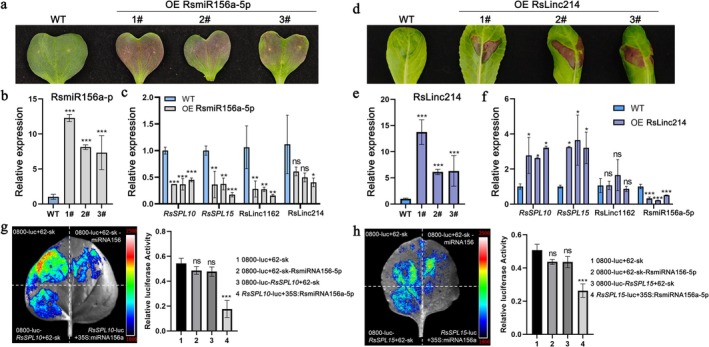
miR156–SPL regulatory module analysis in radish leaves. (a) Leaf phenotypes of wild‐type (WT) and RsmiR156a‐5p overexpression (OE RsmiR156a‐5p) lines. Three independent transgenic lines (1#–3#) are shown. (b) Relative expression levels of RsmiR156a‐5p in WT and OE RsmiR156a‐5p lines determined by qRT‐PCR. (c) Expression analysis of *RsSPL10*, *RsSPL15*, and indicated lncRNAs in WT and OE RsmiR156a‐5p lines. (d) Leaf phenotypes of WT and *RsSPL10* overexpression (OE RsLinc214) lines. (e) Relative expression levels of RsLinc214 in WT and OE RsLinc214 lines. (f) Expression levels of *RsSPL10, RsSPL15*, RsLinc1162, and miR156 in WT and OE RsLinc214 lines. (g, h) Dual‐luciferase reporter assays showing the effects of miR156 on *RsSPL10* (g) and *RsSPL15* (h). Representative luminescence images (left) and quantification of relative luciferase activity (right) are shown.

In order to analyze the function of miR156‐SPL and its targets in radish leaves, transient overexpression of RsmiR156a‐5p in radish leaves was conducted. Compared with the wild type (WT), RsmiR156a‐5p overexpression plants with RUBY tag displayed red color in leaves (Figure 7a). qRT‐qPCR results showed that the expression of RsmiR156a‐5p was significantly upregulated in all three independent overexpression lines (Figure 7b). Overexpression of miR156a‐5p was significantly inhibited the expression levels of *RsSPL10* and *RsSPL15* (Figure 7c). In addition, the expression levels of lncRNAs RsLinc1162 (TCONS_00085696) and RsLinc214 (TCONS_00023439) were decreased in RsmiR156a‐5p overexpression plants. We also overexpressed RsLinc214 plants in radish leaves (Figure 7d). qRT‐qPCR results confirmed that RsLinc214 was significantly upregulated in three independent transgenic lines (Figure 7e). Overexpression of RsLinc214 increased the expression levels of *RsSPL10* and *RsSPL15*, which decreased the expression levels of RsmiR156a‐5p (Figure 7f). Overexpression of RsLinc214 had no significant effect on the expression levels of RsLinc1162. In order to verify the direct regulation of miR156 on *RsSPL10* and *RsSPL15*, transient expression analysis was performed using a dual luciferase reporter system. The results showed that the co‐expression of miR156 significantly inhibited the luciferase activity of *RsSPL10*‐LUC and *RsSPL15*‐LUC reporter genes, as compared with the empty vector control (Figure 7g,h), indicating that miR156 can directly target *RsSPL10* and *RsSPL15* and inhibit their expression.

## 4 Discussion

### 4.1 Differentially Expressed Genes of Flower Development Identified in the Radish

The transition from vegetative to reproductive growth is a crucial event in vegetable crops. The yield and quality of radish were seriously affected by the premature bolting and flowering. Some progress has been made in the identification and function analysis of bolting and flowering in radish. However, the molecular mechanism underlying the flowering transition in radish remains elusive. In this study, 5315 DEmRNAs were identified between vegetative growth and flower stage in radish, which were divided into six clusters by k‐means clustering analysis. In cluster 1, the *RsAGL18*, *RsFT*, and *RsELF3* were differentially expressed between two stages. The *FT* facilitated the transition to flowering and overexpression of *AGL19* resulted in a distinct upregulation of *FT* (Kang et al. 2015; Wickland and Hanzawa 2015). Many flower‐related DGEs were identified in cluster 2, such as *RsSPL15*, *RsSOC1*, *RsSWEET11*, and *RsSWEET12*. The *SPL15* in cooperation with floral integrator *SOC1* promoted flowering (Hyun et al. 2016). The *SOC1* interacted directly with the *AGL24* and *SOC1* expression is upregulated by *AGL24* at the shoot apex at the floral transitional stage (Liu et al. 2008). The expression of *SWEET11* and *SWEET12* was suppressed in WT rosette leaves following fungal inoculation at flowering in Arabidopsis (Le Hir et al. 2015). In cluster 3, the *RsFLC*‐like genes, *RsSPL4*, *RsPIF4, RsAGL18*, *RsELF4*, and *RsFLC5*, were differentially expressed between two stages. The *SPL4* as transcriptional activators was interacted with *FD*, which played an essential role in photoperiodic flowering (Jung, Park, et al. 2016). *PIF4* were directly activated *FT* and showed early flowering phenotype at higher temperatures (Kumar et al. 2012). Mutations in *elf4* caused an early flowering, which were involved in photoperiod perception and circadian regulation (Doyle et al. 2002). In cluster 4, *RsCOL1* was differentially expressed between two stages. It was found that overexpression of *COL1* can shorten the period of two distinct circadian rhythms (Ledger et al. 2001). In cluster 6, the *RsFLC*‐like, *RsAGL18*, *RsEMF2*, *RsCPK6*, and *RsVIN3* were differentially expressed in XHT_VG and XHT_F samples. The *FLC* as a central floral repressor had a central role in regulating the response to vernalization in Arabidopsis (Sheldon et al. 2000). The AGL18 as repressors played important role in the floral transition in Arabidopsis (Adamczyk et al. 2007). Suppression of *EMF2* resulted an early‐flowering phenotypes, indicating that the *EMF2* played a critical role in repression of the reproductive development (Yoshida et al. 2001). It was showed that *CPK6* expressed in shoot apical meristem and directly interacted with *FD* (Kawamoto et al. 2015). The *VIN3* was participated in a transient repression of *FLC* by histone deacetylation (Sung and Amasino 2004). Many flower DGEs associated with reproductive process, multiorganism process, response to hormone were identified, which were consistent with GO enrichment analysis in cluster 1 and cluster 3. These result would provide a data basis to further investigate the molecular mechanism of bolting and flowering in radish.

### 4.2 LncRNAs Are Important for Flower Development in the Radish

Many studies have been indicated that lncRNAs play important roles the regulation of plant growth, development and flower in plant species (Swiezewski et al. 2009; Heo and Sung 2011). A total of 169 DElncRNAs were involved in response to heat stress in radish (Yang, Ma, et al. 2019). Previous studies suggested that *LINC15957* was positively regulated anthocyanin accumulation in radish by transient overexpression and VIGS assays (Tan et al. 2023). However, the comprehensive identification of lncRNAs associated with flower development in radish was not reported.

In this study, 263 DElncRNAs were identified in two stages and the expressions level of lncRNAs was lower than of mRNAs, which was similar to previous reports in *Brassica. rapa* (Huang et al. 2018) and tomato (Cui et al. 2017). LncRNAs could regulate gene expression either in cis or in trans (Fatica and Bozzoni 2014). In this study, 84 and 107 DElncRNAs were found to have potential cis‐ and trans‐regulatory effects on 74 DEmRNA and 96 DElncRNAs, respectively. Two lncRNAs *COOLAIR* (transcribed from the 3′ end of the FLC) and *COLDAIR* (transcribed from first intron of *FLC*) are well‐known to involve in the regulation of *FLC* expression under vernalization (Swiezewski et al. 2009; Heo and Sung 2011). A circadian‐regulated lncRNA *CDF5 LONG NONCODING RNA* (*FLORE*) could function in repressing *CDF1*, *CDF3*, *CDF5* and increasing *FT* expression (Henriques et al. 2017). The intergenic lncRNA (*FLAIL*) represses flowering by reducing mRNA expression of its direct target *LAC8* in Arabidopsis (Jin et al. 2023). Many flower‐related genes have cis‐ and trans‐regulated target genes, such as *SOC1* have potential cis‐regulatory effects on 12 DEmRNAs and one DElncRNAs. Therefore, the lncRNAs and cis‐ and trans‐regulated target genes identified in this study are likely to play a regulatory role in flower development in radish.

### 4.3 MiRNAs Regulated lncRNAs and mRNAs in Flower Development in the Radish

Many studies reported that miRNAs play important roles in flowering by suppressing the expression of the complementary mRNAs of their target genes through either cleavage and/or translational inhibition (Wang et al. 2009; Teotia and Tang 2015). In this study, 85 differently DGEs were negatively correlated with 31 DEmiRNAs, of which miR156, miR399, miR824, miR172, and miR169 are well known to regulate flowering time (Mathieu et al. 2009). Previous studies demonstrated that miR156‐targeted *SPL* transcription factors played a role in regulating the transition from the vegetative phase to the floral phase in Arabidopsis (Wang et al. 2009). The *SPL15* and cooperation with *SOC1* form a complex and cooperate to promotes flowering and target gene activation (Hyun et al. 2016). In this study, miR156a‐5p were negatively correlated with three DEGs and 12 DElncRNAs and differentially expressed in XHT_VG and XHT_F samples. Transient overexpression of miR156a‐5p was significantly inhibited the expression levels of *RsSPL10* and *RsSPL15*. The results showed that the miR156 co‐expressed with *RsSPL10* and *RsSPL15* significantly inhibited the luciferase activity of *RsSPL10* and *RsSPL15* genes, indicating that miR156 can directly target *RsSPL10* and *RsSPL15* and inhibit their expression. These result suggested that miR156a‐5p/SPL module network may play an important role in flower development in radish. It was found that the miR399‐PHO2 module involved in the regulation of flowering time by regulating expression of FLC/SOC1 and TSF (TWIN SISTER OF FT) in Arabidopsis thaliana (Kim et al. 2011). Here, miR399b‐targeted *RsFD* are negatively differentially expressed in two stages. The *FD* preferentially expressed in the shoot apex was cooperated with *FT* to promote flowering (Abe et al. 2005). The miRNA novel‐23 and its targeted gene *RsSOC1* were differentially expressed in two stages. The *SOC1* gene integrates multiple flowering signals derived from photoperiod, temperature, hormone, and age‐related signals for flowering in Arabidopsis (Lee and Lee 2010). These results indicated that the new identified miRNA and targeted gene provide abundant data to validate their function associated with flowering time in radish.

LncRNAs act as precursors or target mimics of miRNA play important roles in regulating miRNAs function. Previous studies suggested that braeTM160‐1 and bra‐eTM160‐2 as potential eTMs for bra‐miR160‐5p were involved in pollen development in *Brassica rapa* (Huang et al. 2018). In present study, 32 differentially expressed DEmiRNAs were negatively correlated with 87 DElncRNAs in 134 miRNA‐target modules. Many flower related miRNA target DElncRNAs were identified, including miR156‐5p–targeted 12 DElncRNAs and miR172b‐5p–targeted one DElncRNAs. The novel‐23–targeted three DElncRNAs were detected. Transient overexpression of miR156a‐5p was significantly inhibited the expression levels of lncRNAs RsLinc1162 and RsLinc214. According to the targets mRNA and lncRNA of miRNA, five mRNA‐lncRNA‐miRNA regulatory network were constructed. The comprehensive identification and analysis of mRNA‐lncRNA‐miRNA networks associate with flowering transition will provide data reference to investigate the regulation mechanism of flowering time in radish.

## 5 Conclusions

In this study, we conducted a comprehensive characterization of mRNAs, lncRNAs, and miRNAs at the vegetative growth and flowering stages. A total of 5315 DGEs, 263 DElncRNAs, and 38 DEmiRNAs were identified. Many flower DGEs associated with reproductive process, multiorganism process, and response to hormone were identified by cluster analysis. In total, 202 DElncRNAs and 257 DElncRNAs were found to have potential cis‐ and trans‐regulatory effects on 572 DEmRNAs and 3902 DGEs, respectively. A total of 93 and 82 DEGs were predicted as putative targets of 31 DEmiRNAs and 29 DEmiRNAs, respectively. Five mRNA‐lncRNA‐miRNA regulatory pairs involved in flowering time regulation were constructed. These findings provide valuable candidates DGEs, DElncRNAs, and DEmiRNAs for further exploring the complex regulatory mechanism of bolting and flowering in radish.

## Author Contributions

Xiaobo Luo and Wanping Zhang designed the experiments. Xiaobo Luo performed the experiments and analyzed the data. Linjun Wu, Yueyue Jin, and Yadong Li performed the qRT‐PCR experiments. Xiaobo Luo and Wanping Zhang drafted and revised the manuscript. All authors have read and agreed to the published version of the manuscript.

## Funding

This research was funded by the Guizhou Provincial Science and Technology Projects (Qiankehe [2021] general 213), and the National Natural Science Foundation of China (31960598), Guizhou Highland Specialty Vegetable Green Production Science and Technology Innovation Talent Team (Qiankehe Platform Talent–CXTD [2022]003).

## Conflicts of Interest

The authors declare no conflicts of interest.

## Supporting information




**Table S1:** The qRT‐PCR primers used in this study.
**Table S2:** The statistics of RNA‐seq in two stages.
**Table S3:** The information of DElncRNAs idengtified in this study.
**Table S4:** The expression information of DEGs identified in this study.
**Table S5:** The significant GO terms of all DEGs identified in this study.
**Table S6:** The significant KEGG terms of all DEGs identified in this study.
**Table S7:** The statistics of small RNA sequencing in two stages.
**Table S8:** The expression information of miRNAs identified in this study.
**Table S9:** The cis regulated DGEs of DElncRNAs.
**Table S10:** The trans‐regulated DGEs of DElncRNAs.
**Table S11:** The DEmiRNAs‐targeted DEmRNAs in two stages.
**Table S12:** The DEmiRNAs‐targeted DElncNAs in two stages.

## Data Availability

The RNA‐seq raw data have been deposited with NCBI with the BioProject Number PRJNA1035533, and other data presented in this study are available in the Supplementary Material of this article.
